# Pulmonary Hydatid Cyst in Children: A Single-Institution Experience

**DOI:** 10.7759/cureus.26670

**Published:** 2022-07-08

**Authors:** Osman Hakan Kocaman, Tansel Günendi, Osman Dere, Mustafa Erman Dörterler, Mehmet E Boleken

**Affiliations:** 1 Pediatric Surgery, Harran University Faculty of Medicine, Şanlıurfa, TUR; 2 Radiology, Harran University Faculty of Medicine, Şanlıurfa, TUR

**Keywords:** pulmonary hydatidosis, cyst rupture, capitonnage, children, echinococcus

## Abstract

Objective: Hydatid cysts can occur in any place such as the liver, lung, spleen, kidney, brain, and soft tissue. Pulmonary hydatid cysts are more prone to rupture than liver hydatid cysts. In this study, we aimed to present the demographic characteristics, clinical symptoms, radiological findings, surgical findings, type of surgery performed, and postoperative complications of patients with pulmonary hydatid cysts.

Materials and method: The files of 94 patients who were operated on for pulmonary hydatid cysts in our clinic between January 2011 and October 2021 were retrospectively analyzed. The patients were divided into two groups: ruptured pulmonary hydatid cysts and non-ruptured pulmonary hydatid cysts.

Results: A total of 120 pulmonary hydatid cysts were detected in 94 patients who were operated on for pulmonary hydatid cysts. Cyst rupture was detected in 63 (52.5%) patients. Rupture was found significantly higher in cysts with a diameter of <10 cm (p=0.005). Complaints of fever and hemoptysis were found significantly higher in the ruptured group. Pneumothorax was detected in six patients after the operation with an average of two months, one of which was the patient who underwent video-assisted thoracoscopy (VATS).

Conclusion: Pulmonary hydatid cyst should be kept in mind in children presenting with lower respiratory tract symptoms in regions where echinococcosis is endemic. Parenchyma-sparing methods should be the first choice in the management of pulmonary hydatid cysts. Patients who develop early postoperative complications should also be followed closely for late impediments.

## Introduction

Hydatid cyst is a zoonotic disease caused by echinococcal tapeworms. Traditionally, it has been reported in Central Asia, the Middle East, the Mediterranean region, East Africa, South America, New Zealand, and Australia due to its endemic nature related to animal husbandry [1].

Orally ingested eggs pass through the mucosa of the upper intestinal tract and pass into the portal venous system. Later, they transform into larval form in the terminal organ where the eggs are held [2]. Hydatid cysts can occur in any place such as the liver, lung, spleen, kidney, brain, and soft tissue. While liver involvement is more common in adults, lung involvement is encountered more commonly in children [3]. Hydatid cysts grow faster in the lung than in the liver due to the elastic structure of the lung, and they can reach giant sizes and be invasive to most of the lobe [4]. Another mechanism is probably negative intrathoracic pressure, which may cause rapid growth of the pulmonary cyst [5]. Pulmonary hydatid cysts are more prone to rupture than liver hydatid cysts [6]. Children with pulmonary hydatid cysts are mostly diagnosed with respiratory symptoms such as cough, hemoptysis, chest pain, shortness of breath, and fever or by incidental detection.

The treatment of pulmonary hydatid cysts is surgery. Pulmonary cystotomy and capitonnage are the most frequently preferred surgical methods because they protect the lung tissue. However, lobectomy and even pneumonectomy are recommended in selected cases when they cause severe parenchymal damage or in complications such as bronchiectasis, chronic abscess, or severe bleeding [7,8].

In this study, we aimed to present the demographic characteristics, clinical symptoms, radiological findings, surgical findings, type of surgery performed, and postoperative complications of patients with pulmonary hydatid cysts.

## Materials and methods

After obtaining the approval of the local clinical research ethics committee (Ethics Committee Decision Number 21.21.19), the files of 94 patients who were operated on for pulmonary hydatid cysts in our clinic between January 2011 and October 2021 were retrospectively analyzed. An informed consent form was obtained from the parents of patients who were operated on. All procedures performed in this study involving human participants complied with the ethical standards of the institutional and/or national research committee and the 1964 Declaration of Helsinki and its subsequent amendments or comparable ethical standards. Patients’ symptoms, radiological findings, hemagglutination tests, surgical findings, type of operation method, length of hospital stay, and complications were investigated.

**Figure 1:**
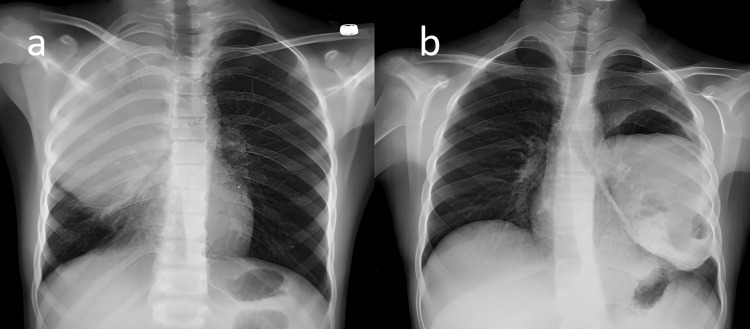
Chest X-ray image of ruptured and non-ruptured pulmonary hydatid cysts (a) Chest X‑ray showing a giant spherical cystic mass (hydatid cyst) displayed in the upper part of the right lung. (b) Chest X-ray showing a cystic mass showing air-fluid level in the lower lobe of the left lung (ruptured hydatid cyst).

**Figure 2:**
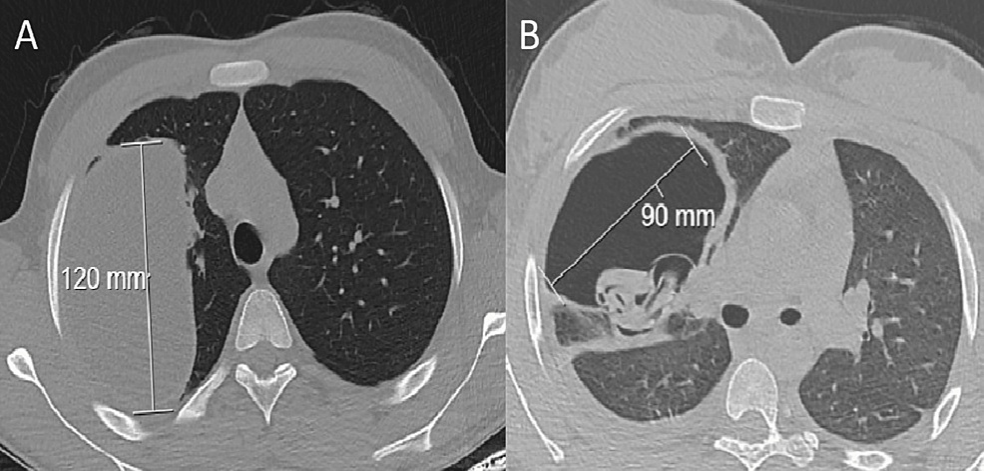
CT image of ruptured and non-ruptured pulmonary cysts (A) Axial chest CT image in the parenchymal window showing an uncomplicated hydatid cyst of the right lung. (B) Axial chest CT image in the parenchymal window showing a ruptured hydatid cyst of the right lung. Air in the cyst caused by bronchial rupture of the cyst and germinative membrane (water lily sign) can be seen.

The diagnosis of pulmonary hydatid cyst was established using chest X-ray (Figure 1) and thorax computed tomography (CT). Routine abdominal ultrasound (US) was performed in patients diagnosed with pulmonary hydatid cysts to see if there was any intra-abdominal involvement. Standard laboratory tests were requested from all patients, but indirect serological tests were not routinely requested. The patients were divided into two groups, ruptured and non-ruptured, according to preoperative thorax CT findings (Figure 2).

Surgical intervention was performed with posterolateral thoracotomy for unilateral lung hydatid cysts. In cases with bilateral lung involvement, posterolateral thoracotomy was performed first on the ruptured side in the existence of a cyst rupture or on the side with a large cyst diameter, and a separate operation was performed for the contralateral lung preferably one or two months later. Transdiaphragmatic liver hydatid cysts were operated on in the same session for cysts in the liver dome along with the right lung. Our routine surgical technique for lung hydatid cysts is parenchyma-sparing cystotomy with bronchial fistula closure and capitonnage. Hypertonic saline sponges were placed around the operation site to prevent a possible rupture of the cyst from infecting the surrounding tissues. After injecting hypertonic saline into the cavity and aspirating the cyst contents, the intact germinative membrane was removed. Bronchial fistulas, if any present, were closed with vicryl sutures, and the cyst wall was then capitonated. A thorax tube was inserted into the pleural cavity.

However, segmentectomy or lobectomy was performed in cases where the cyst had severely destroyed the infected lung tissue. Preoperative albendazole was not given; however, in the postoperative period, it was given at 15 mg/kg/day in two equal doses for a duration of six months. The patients were reevaluated on follow-up with chest X-rays at one month, three months, six months, one year, and two years after surgery, and additional thorax CT was taken as per necessity.

The SPSS Statistics for Windows version 23.0 (IBM Corp., Armonk, NY, USA) was used as the statistical analysis program. Descriptive statistics (mean, standard deviation (SD), frequency, and percentage) were used for the demographic and clinical characteristics. Student’s t-test was used for comparing two groups of continuous data, which are both normally distributed. Categorical data were compared using the chi-square test. A p-value of <0.05 was considered statistically significant.

## Results

Overall, 120 pulmonary hydatid cysts were detected in 94 patients who were operated on for pulmonary hydatid cysts. Of the patients, 54.3% were male and 45.7% were female. The mean age of the patients was 8.95±3.88 years. The most common presenting symptom was cough with 63.8%. While only one patient presented with an anaphylactic shock, three patients underwent emergency thoracotomy due to severe respiratory distress. In the thoracic CT examination, a lung hydatid cyst of >10 cm was detected in 28.3% of the patients.

Right lung involvement was detected in 43.7% of the patients, while bilateral lung involvement was detected in 16%. In 30 patients, liver or spleen involvement was detected in addition to the lung. Indirect hemagglutination assay (IHA) was reported as negative in 21 of 34 patients. Prolonged air leak was (>7 days) the most common postoperative complication in seven (7.4%) patients. The mean hospital stay was found to be 8.76±4.80 days. The detailed demographic characteristics and clinical findings of the patients are shown in Table 1.

**Table 1:** Patient demographics and clinical characteristics IHA: indirect hemagglutination assay

Characteristics	Number (94)	Percentage (%)
Age (mean±SD)	8.95±3.88	
Gender		
Female	43	45.7%
Male	51	54.3%
Presenting symptoms		
Cough	60	63.8%
Chest pain	23	24.5%
Fever	16	17%
Hemoptysis	13	13.8%
Abdominal pain	7	7.4%
Shortness of breath	5	5.3%
Incidental	5	5.3%
Clear fluid expectoration	2	2.1%
Daughter vesicle expectoration	1	1.1%
Anaphylaxis	1	1.1%
Lung side		
Right	41	43.7%
Left	38	40.4%
Bilateral	15	16%
Other organ involvement	30	31.9%
Liver	29	
Spleen	2	
IHA		
Negative	21	61.8%
Positive	13	32.2%
Early complications		
Prolonged air leak (>7 days)	7	7.4%
Atelectasis	4	4.3%
Late complication		
Pneumothorax	6	6.4%

When the thorax CT of the patients were examined, cyst rupture was detected in 63 (52.5%) patients. While cysto-bronchial fistula was detected in most patients, cysto-pleural fistula was detected in only six patients. Of the six patients with cysto-pleural fistula, two had pneumothorax, three had hydropneumothorax, and one had pleural effusion in whom a thoracic tube was inserted in another medical center and was followed up for two weeks. The patients whose complaints did not resolve were referred to us because of the cavitary lesion seen in the thorax CT scans of the patients.

While more than one unilateral pulmonary cyst was detected in three patients, multiple lung involvement was detected in only two patients. Pulmonary hydatid cysts were found most frequently in the left lower lobe (27.5%) and least frequently in the right upper lobe (14.9%). In 30 patients, additional organ involvement other than lung was detected.

No significant difference was found in terms of age, gender, and cyst localization in the comparison of both groups (p>0.05). IHA positivity was found to be significantly higher in ruptured cysts (p=0.02). Rupture was found significantly higher in cysts with a diameter of <10 cm (p=0.005). Although the length of hospital stay is not statistically significant, it is 1.5 days longer in rupture patients (p=0.096). Complaints of fever and hemoptysis were found significantly higher in the first group (p=0.008 and p=0.009, respectively), and no relationship was found between cyst rupture and other symptoms (p>0.05). The relationship between the demographic and clinical characteristics of the patients and the rupture of the cyst is shown in Table 2, and the relationship between the admission symptoms of the patients and the rupture of the cyst is shown in Table 3.

**Table 2:** Association between patients’ demographics and clinical characteristics and cysts’ rupturing IHA: indirect hemagglutination assay *p<0.05

Features	Options	Ruptured cyst (group 1) (n=63, 52.5%)	Unruptured cyst (group 2) (n=57, 47.5%)	p-value
Age	-	9.00±3.86	8.92±3.97	0.914
Sex	Female	25	18	0.208
	Male	23	28	
Cyst localization	Right upper lobe	10	7	0.921
	Right middle lobe	14	13	
	Right lower lobe	11	10	
	Left upper lobe	12	10	
	Left lower lobe	16	17	
Liver involvement	Negative	37	40	0.235
	Positive	24	19	
IHA	Negative	6	15	0.020^*^
	Positive	9	4	
Cyst dimensions	<10 cm	51	35	0.005^*^
	>10 cm	12	22	
Complication	Negative	54	52	0.649
	Positive	6	5	
Length of hospital stay (days)	-	9.56±5.40	7.91±3.98	0.096

**Table 3:** Relationship between presenting symptoms and cyst’s rupturing *p<0.05

Symptoms		Ruptured cyst	Unruptured cyst	p-value
Cough	Yes	34	26	0.149
	No	14	20	
Clear fluid expectoration	Yes	1	1	0.976
	No	47	45	
Fever	Yes	13	3	0.008^*^
	No	35	43	
Hemoptysis	Yes	11	2	0.009^*^
	No	37	44	
Shortness of breath	Yes	1	4	0.153
	No	47	42	
Chest pain	Yes	9	14	0.188
	No	39	32	
Incidental	Yes	0	5	0.019^*^
	No	41	48	
Abdominal pain	Yes	2	5	0.216
	No	46	41	

Cystotomy and capitonnage were performed on 112 of 120 pulmonary hydatid cysts, and one of them was performed using the video-assisted thoracoscopic surgery (VATS) method. Segmentectomy or lobectomy was performed in eight pulmonary hydatid cysts with multiple cysts in a single lobe and severe parenchymal destruction. The mean follow-up period of the patients was 2.7 years. Early postoperative complications were detected in 11.7% of the patients, and prolonged air leakage was the most common (7.4%). No additional surgical intervention was performed for early postoperative complications. Pneumothorax was detected in six patients after the operation with an average of two months, one of which was the patient who underwent VATS. Four patients were treated with tube thoracostomy, one patient underwent re-thoracotomy + fistula repair, and one patient underwent re-thoracotomy + segmentectomy. No mortality or recurrence was detected in the patients.

## Discussion

Hydatid cyst disease is still a public health problem in developing countries, especially in endemic ones. Approximately 10%-20% of hydatid cysts are seen in childhood [6]. Lung cysts usually develop with the hematological spread of larvae through the hepatic sinusoids, and more rarely, they occur as a result of the spread of parasites into the lymphatic circulation [6]. Contrary to adults, hydatid cysts in children occur in the lung in 64% of cases and in the liver in 28% [9]. Because of the compressibility of the lung tissue, its higher vascularity, and lower negative pressure, pulmonary hydatid cysts more easily become symptomatic [3]. Because of the high compliance and flexibility of the lungs, hydatid cysts can grow up to more than 5 cm per year [10].

In previous studies, the rate of bronchial rupture varied between 21.1% and 53.1% [11,12]. In our study, we found bronchial rupture at a rate of 52.5%, which is consistent with the literature. We detected cysto-bronchial fistula in four (3.3%) patients who underwent emergent thoracotomy. A ruptured pulmonary hydatid cyst should be kept in mind in patients presenting with anaphylaxis or acute respiratory distress without any known disease.

Previous studies have shown that pulmonary hydatid cysts are more common in males [8]. Although the number of male patients was high in our study, statistical significance was not found; this was valid both for the ruptured and non-ruptured groups. This is attributed to the fact that both females and males are almost equally affected, as rural areas with livestock farming are abundant in our region. The onset of symptoms is often seen later in adulthood because hydatid cysts grow slowly in other organs [2]; pulmonary hydatid cysts show up at an earlier time since they may tend to grow faster. They present in childhood with symptoms such as cough, hemoptysis, chest pain, fever, and shortness of breath. In our study, the most common complaints were cough, chest pain, fever, and hemoptysis. When a comparison between the groups was made, fever and hemoptysis were significantly higher in the ruptured group. This may be attributed to the systemic and local inflammation after rupture.

Ngcobo et al. found 52.4% IHA positivity in ruptured hydatid cysts and 41.2% in non-ruptured hydatid cysts, but they did not find a statistical difference between the two groups [1]. Özdemir et al. found statistically significantly higher IHA positivity in ruptured hydatid cysts in a study of 649 patients [13]. In our study, we found statistically significant IHA positivity in ruptured patients. We interpret this occurrence as a systemic response to the cyst rupture.

Pulmonary hydatid cysts with a diameter larger than 10 cm are called giant cysts [14]. Giant pulmonary cysts have been reported with a rate of 15%-31% in pediatric series [9]. We determined it as 17.5% in our study. According to some authors, as the diameter of the cyst increases, the rupture rate also increases [8,15]. On the other hand, Kuzucu et al. did not find a significant difference between cyst diameter and rupture [16]. On the contrary, Burgos et al. reported an enlargement in cyst size as a negative risk factor for rupture [17]. In the study by Akgul Ozmen et al., the mean cyst diameter was significantly larger in the unruptured group than in the ruptured group [18]. In our study, 89.9% of ruptured cysts were smaller than 10 cm, which was statistically significant. This may be due to the shrinkage of the cyst after rupture. Previous radiological examinations of these patients could not be reached because they were admitted to our clinic after the cyst ruptures without any earlier radiological follow-ups. For this reason, one cannot be sure whether the cysts are ruptured because they were small or whether their diameters are reduced after the rupture.

Although pulmonary hydatid cysts can infest all lobes of the lung, it has been reported that they are found more commonly and especially in the right lower lobe [19]. However, in the study by Hamouri et al., they observed that both ruptured and non-ruptured cysts were more prevalent in the left lower lobe [20]. In our study, cysts were detected in the left lower lobe, right middle lobe, left upper lobe, right lower lobe, and right upper lobe, in order of frequency. The rupture rates of the cysts were also similar.

The main treatment of pulmonary hydatid cysts is conventional surgery. The procedure aims to remove all parasitic substances and close the possible existing bronchial fistulas while preserving the maximum lung tissue, especially in children. Many techniques have been described for surgical treatment, such as open enucleation, peri-cystectomy, cystotomy and capitonnage, cystotomy without capitonnage, segmental resection, and lobectomy [2]. Each method has its own advantages and disadvantages. Any of these methods can also be performed with thoracoscopy, but the complication rate was found to be higher in pulmonary hydatid cysts [21]. He et al. found only one bronchopleural fistula in 12 patients who were performed on with cystotomy without capitonnage [22]. However, in the study of Ksia et al. that included 136 patients, they compared the groups of capitonnage and non-capitonnage patients, and pneumothorax was determined statistically lower [9].

In our patient group, we performed cystotomy + capitonnage in 93.3% of 120 cysts. We detected multiple hydatid cysts in two patients who underwent segmentectomy or lobectomy that involve a single lobe. Multiple fistulas in a single cyst were encountered in two patients, and an abscess was seen in the single cyst along with the surrounding lung tissue in four patients. Although early and late complications were not observed in patients who underwent segmentectomy or lobectomy, prolonged air leak or atelectasis was found in 11.7% of patients who underwent cystotomy and capitonnage, which also did not require any additional surgical intervention. Two of the patients who developed early postoperative complications were first followed up conservatively, and then, a re-thoracotomy was performed accordingly.

Preoperative albendazole treatment in pulmonary echinococcosis is not preferred because of cyst wall rupture for years [23,24]. Postoperative use of albendazole for three or six months has become routine worldwide. Thus far, we also have given our patients albendazole for six months and encountered no recurrence.

The limitations of this study are its retrospective nature and the absence of previous radiological images before cyst rupture.

## Conclusions

Pulmonary hydatid cyst should be kept in mind in regions where echinococcosis is endemic. Therefore, in pediatric patients presenting with lower respiratory tract symptoms such as fever, cough, and hemoptysis, further imaging methods such as thorax CT should be performed. Parenchyma-sparing methods should be the first choice in the management of pulmonary hydatid cysts, which requires sophisticated knowledge and experience in segmentectomy and lobectomy to prevent intraoperative and postoperative complications. Postoperatively, two doses of albendazole at 15 mg/kg/day is effective in preventing recurrence. Patients who develop early postoperative complications should also be followed up closely for late impediments.
